# Increased sulfur-containing amino acid content and altered conformational characteristics of soybean proteins by rebalancing 11S and 7S compositions

**DOI:** 10.3389/fpls.2022.828153

**Published:** 2022-09-02

**Authors:** Biao Wang, Da Teng, Cunhao Yu, Luming Yao, Xiaohong Ma, Tianlong Wu

**Affiliations:** ^1^School of Agriculture and Biology, Shanghai Jiao Tong University, Shanghai, China; ^2^Shanghai Collaborative Innovation Center of Agri-Seeds, Shanghai, China; ^3^College of Agriculture, Northeast Agricultural University, Harbin, China

**Keywords:** sulfur-containing amino acids, conformational characteristics, soybean proteins, RNA interference, 11S glycinin, 7S globulin

## Abstract

Soybean proteins are limited by their low contents of methionine and cysteine. Herein, 7S globulin accumulation was reduced using RNA interference to silence *CG-β-1* expression, and the content of the A2B1a subunit was largely increased under the soybean seed-specific oleosin8 promoter. The results showed that the sulfur-containing amino acid content in soybean seeds drastically improved, reaching 79.194 nmol/mg, and the 11S/7S ratio had a 1.89-fold increase compared to the wild-type acceptor. The secondary structures of 11S globulin were also altered, and the β-sheet content increased with decreasing β-turn content, which was confirmed by Fourier transform infrared spectroscopy, Raman spectroscopy and circular dichroism analysis. Our findings suggested that raising the accumulation of 11S glycinin at the expense of reducing the content of 7S globulin is an attractive and precise engineering strategy to increase the amount of sulfur-containing amino acids, and soybean proteins with A2B1a subunits of 11S isolates improved, and β-subunits of 7S fractions reduced simultaneously might be an effective new material for food production.

## Introduction

Soybean [*Glycine max* (L.) Merr.] is a remarkable crop that serves as an excellent protein source for both humans and livestock (Krishnan and Jez, 2018). In contrast to the high protein content in soybeans, the sulfur-containing amino acids methionine and cysteine are deficient in monogastric diets and rations. Methionine cannot be synthesized in humans and monogastric animals and is considered an “essential” amino acid, and cysteine is a “conditionally” essential amino acid for animals that can convert methionine into cysteine *in vivo* (Brosnan and Brosnan, 2006). There is a negative influence from an inadequate intake of sulfur-containing amino acids on the growth and development of animals. Therefore, it is therefore crucial to enhance the concentrations of these two sulfur-containing amino acids in soybeans (Imsande, 2001). Considerable efforts have been directed toward improving the content of sulfur-containing amino acids in soybean seeds, and multiple approaches, including traditional breeding and biotechnological methods, have been proposed to realize high concentrations of methionine and cysteine in proteins (Zhang et al., 2014). In the early stages of research, breeding methods that included the use of spontaneous or induced soybean mutants were used to increase the content of sulfur-containing amino acids (Paek et al., 1997, 2000). Imsande (2001) selected several methionine-overproducing genetic lines whose sulfur-containing amino acid concentrations were approximately 20% higher than those of the parent lines.

Biotechnological approaches have also been undertaken to increase the sulfur-containing amino acid content of soybean seeds. Several methionine-rich heterologous proteins have been overexpressed in transgenic soybean. For example, sulfur-rich zeins from maize have been expressed in transgenic soybean plants (Dinkins et al., 2001; Li et al., 2005; Kim and Krishnan, 2019), and even the *de novo* synthetic protein MB-16 has been introduced into soybean to improve protein quality (Zhang et al., 2014). Recent experiments have been developed to increase the sulfur-containing amino acid content of seeds by enhancing the expression of enzymes involved in the sulfur assimilatory and cysteine/methionine biosynthesis pathways. Overexpression of ATP sulfurylase increased the protein-bound cysteine and methionine contents in transgenic soybean seeds by 37–52% and 15–19%, respectively (Kim et al., 2020). A cytosolic isoform of O-acetylserine sulfhydrylase (OASS) has been introduced into soybean and could increase protein-bound cysteine levels by 58–74% compared with those of nontransformed wild-type seeds, and a 22–32% increase in free cysteine levels was also observed. Furthermore, the accumulation of a cysteine-rich protein, a Bowman-Birk protease inhibitor, markedly increased in transgenic soybean plants (Kim et al., 2012). The feedback-insensitive cystathionine γ-synthase obtained from *Arabidopsis* was also introduced into soybean, and the accumulation of methionine increased by 4.8-fold and 2.3-fold in seeds of transgenic Zigongdongdou and Jilinxiaoli 1 lines, respectively (Yang et al., 2018).

The major proteins stored in soybean seeds are β-conglycinin (a 7S globulin of 180 kDa) and glycinin (an 11S globulin of approximately 360 kDa), which account for 30–40% of the total weight (Nishinari et al., 2014). 7S β-conglycinin consists of three subunits, α, α’ and β, with molecular weights of 67 kDa, 71 kDa and 50 kDa, respectively (Wu et al., 2016). The mature β-subunit of combined soybean seed storage proteins is composed of approximately 416 amino acid residues and lacks both methionine and cysteine (Paek et al., 1997). 11S glycinin is composed of six nonrandomly paired acidic and basic peptides. The acidic A polypeptide (37–42 kDa) and basic B polypeptide (17–20 kDa) are linked together by a disulfide bond (Wu et al., 2017). Soybean 11S globulin contains five major subunits, A1aB1b, A1bB2, A2B1a, A3B4, and A5A4B3, and has three to four times more sulfur-containing amino acids than 7S β-conglycinin does. A2B1a (encoded by *gy2*) is the most abundant Met+Cys and is present in all commercial soy protein isolates (Sui et al., 2021).

The specific units of protein secondary structure, including α-helix, β-sheet, β-turn, and random coil, are vital for the rigidity and flexibility of protein (Su et al., 2015). Native soybean glycinin and β-conglycinin are a combination of different isoforms with high heterogeneity, and at the tertiary level, the conformational stability/flexibility of their subunits or polypeptides is difficult to evaluate; in fact, each isoform possesses a quaternary structure. By comparison, the secondary structure in soy glycinin and β-conglycinin is highly ordered and preserved due to the strong restrictions from the presence of their quaternary structure (Tang, 2017). Globulins from soybean typically possess low levels of α-helices and very high β-sheet secondary structures, and they are often considered to be β-type proteins (Marcone, 1999). The conformational characteristics of soybean proteins are complicated by complex mixtures of different constituents with different structural and physicochemical properties. The properties and structural characteristics of soy proteins are highly dependent on the composition and proportion of 7S and 11S globulins (Tezuka et al., 2004; Tang, 2017). The β-sheet structure was most important for its significant role in denaturation of 7S globulin and subsequent formed aggregates and even in denaturation of 11S globulin, and biological structural-functional relationships are usually associated with protein applications (Wang et al., 2014).

In this study, the strong seed-specific promoters of soybean oleosin and α subunits were introduced to enhance the expression of *gy2* and to silence the *CG-β-1* gene by RNA interference, which sharply increased the content of sulfur-containing amino acids and the 11S/7S ratio in soybean seeds. Moreover, the soybean proteins with the A2B1a subunit of 11S isolates improved, and the β-subunit of 7S fractions reduced simultaneously, which would also alter conformational characteristics and supply a new formula for food products.

## Materials and methods

### Construction of vectors

**Figure 1:**
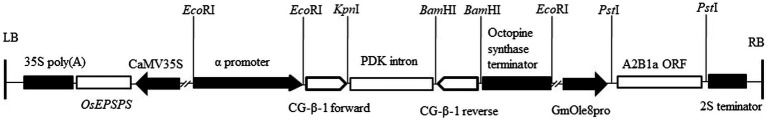
Schematic diagram of pCambia1300-OsEPSPS-RNAi-GmOle8pro-A2B1a. The soybean transformation construct contains the A2B1a subunit coding region (A2B1a ORF) under the control of the soybean oleosin8 promoter (GmOle8pro) and the 3′-end noncoding region of 2S albumin (2S terminator). The RNAi cassette under the control of the soybean α-promoter of β-conglycinin and the octopine synthase terminator, together with 302 bp fragments obtained from sequences of *CG-β-1* gene forward and reverse inserted side of PDK intron, respectively. The *OsEPSPS* gene under the control of the cauliflower mosaic virus 35S promoter (CaMV35S) and 35S poly(A) terminator acted as a selective marker. *Eco*RI, *Kpn*I, *Bam*HI and *Pst*I restriction sites are shown on the vector. LB, left border; RB, right border.

pCambia1300-OsEPSPS-GmOle8pro was gifted by Dr. Zhu (Zhou et al., 2006). For β subunit of β-conglycinin silencing, 302-bp coding *CG-β-1* sequences were selected and amplified by high-fidelity *Taq* enzymes and then ligated into a pHANNIBAL vector in opposite orientations on either side of a PDK intron (Wesley et al., 2001). To more accurately interfere with the β subunit gene and reduce its influence on agronomic traits, the CaMV35S promoter was substituted with a seed-specific promoter from the α subunit of β-conglycinin of soybean. This *CG-β-1* RNAi construct was then cloned into the pCambia1300-OsEPSPS-GmOle8pro plant expression vector to produce pCambia1300-OsEPSPS-RNAi-GmOle8pro. For overexpression of 11S glycinin containing high-sulfur-containing amino acids, the coding sequence of the A2B1a subunit obtained from soybean cultivar ‘Tianlong No. 1’ was cloned into the vector pCambia1300-OsEPSPS-RNAi-GmOle8pro to form pCambia1300-OsEPSPS-RNAi-GmOle8pro-A2B1a (Figure 1). The newly constructed plant expression vector was subsequently inserted into *Agrobacterium* strain EHA105 for transformation of soybean cotyledons.

### Soybean cotyledon transformation

Transgenic soybean events were performed using a modified cotyledon transformation protocol (Du et al., 2016). Briefly, *Agrobacterium tumefaciens* strain EHA105 was chosen for the soybean cultivar ‘Tianlong No. 1’, cultured to an OD_600_ of 0.8–1.0, centrifuged and then suspended in cocultivation media (CCM liquid: 1/10 B5 salts, 30 g/l sucrose, 20 mmol/l MES, 1.67 mg/l BAP, 0.25 mg/l GA3 and 200 μmol/l acetosyringone) to an OD_600_ of 0.4–0.5. The soybean seeds were surface sterilized for 16 h using chlorine gas and germinated overnight on sterile filter paper. The cotyledonary nodes were cut into 5–7 slices vertical to the axis using a blade, dipped into the coculture suspension for 30 min at 28°C and then transferred onto solid cocultivation media (solid: CCM liquid plus 5 g/l agarose, 0.4 g/l L-cysteine, 0.154 g/l DTT, and 0.158 g/l nathiosulfate) for 5 days at 25°C under dark conditions. After cocultivation, the infected explants were washed in washing media (B5 salts, 30 g/l sucrose, 3 mmol/l MES, 1.67 mg/l BAP, 500 μg/ml carbenicillin and 50 μg/ml cefotaxime) and subsequently cultured on SI media (washing media plus 3.5 g/l phytagel) for 2 weeks at 25°C under a 16 h light and 8 h dark photoperiod. The explant tissues were then transferred to fresh SI media supplemented with 8 mg/l glyphosate for an additional 14 days. The differentiating explants were cut and transferred to SE media (MS salts, 30 g/l sucrose, 3.5 g/l Phytagel, 3 mmol/l MES, 5 mg/l asparagine, 10 mg/l pyroglutic acid, 0.1 mg/l IAA, 0.5 mg/l GA3, 1 mg/l zeatin riboside, 500 μg/ml carbenicillin and 50 μg/ml cefotaxime) to initiate shoot elongation with 8 mg/l glyphosate every 2 weeks. To initiate rooting, each elongated shoot (>3–4 cm) was cut and then transferred to rooting media (MS salts, 20 g/l sucrose, 3.5 g/l phytagel, 3 mmol/l MES and 0.5 mg/l IBA). The rooted plantlets were subsequently transplanted into soil and grown in a greenhouse. T_0_ plants were identified *via* PCR and sprayed with 10 ml/l glyphosate. Each generation of positive plants was grown and selected by screening 10 ml/l glyphosate again, and homozygous T_5_ transgenic plants were further analyzed.

### Real-time RT–PCR analysis

Total mRNA was isolated from soybean seeds at the R6 stage using TRIzol reagent (Sangon Biotech, Cat # B610409) and a Spin Column Plant Total RNA Purification Kit (Sangon Biotech, Cat # B518661) and then treated with RNase-free DNase enzyme (Sangon Biotech, Cat. # B300066). For real-time RT–PCR analysis, 1 μg of total purified mRNA was used for cDNA synthesis (Sangon Biotech, AMV Reverse Transcriptase Kit, B600020). Amplifications were performed in a total volume of 10 μl consisting of SGExcel Ultra SYBR Mixture (Sangon Biotech, B110032), primer pairs (each at 0.2 μmol/l), and cDNA (10 ng of starting mRNA), and the soybean *actin* gene (GenBank: accession: V00450.1) was used as the control. The samples were amplified for 40 cycles, starting with an initial activation step of 3 min at 95°C, followed by denaturation for 10 s at 95°C, annealing for 10 s at 55°C and extension for 15 s at 72°C. At the end of PCR amplification, the temperature was increased from 50°C to 99°C at a rate of 0.2°C s^−1^, and melting curves were constructed based on the fluorescence intensity. The primers used for qPCR are listed in Supplementary Table S1. The relative expression levels of the genes were calculated using the 2^−ΔCq^ method (Schmittgen and Livak, 2008).

### ELISA assay

Soy protein isolates (SPIs) were obtained from defatted soybean flakes according to Thanh and Shibasaki’s protocols (Thanh and Shibasaki, 1976). The extracted total protein was resolved in sample buffer containing 62.5 mmol/l Tris–HCl, pH 6.8, 8 M urea, 15% SDS, 20% glycerol and 5% 2-mercaptoethanol, sonicated for 5 min, boiled for 5 min, and then centrifuged at 10,000× *g* for 10 min (Moriyama et al., 2005). The obtained clear supernatant was subsequently quantified using ELISA kits (AMEKO, Cat# AE98502G for glycinin, AE98605G for β-conglycinin) according to the manufacturer’s protocol. Afterward, 100 μl of peroxidase substrate TMB solution was added to each well, and the reaction was terminated by the addition of 4 M H_2_SO_4_ solution (50 μl/well). The color change was measured spectrophotometrically at a wavelength of 450 nm. Calibration curves were then generated by plotting the optical density (OD) values against different gradient concentrations of standard samples.

### Gel permeation chromatography

Gel permeation chromatography was performed using an EcoSEC HLC-8320 GPC system (Japan). Soybean protein isolate (consisting of 0.2% protein, w/v) was dissolved in DI water and filtered through a Millipore membrane (0.45 μm). The following conditions were used: injection volume, 2.0 μl; flow rate, 0.5 ml/min; elution solvent 1, 35 mm phosphoric potassium buffer (pH 7.6) including 0.1 mol/l sodium chloride; and elution solvent 2, 35 mm phosphoric potassium buffer (pH 7.6) including 0.1 mol/l NaCl and 6.0 M urea. The total elution time was 20 min, and the elution absorbance was measured at a wavelength of 280 nm. All the data were recorded and analyzed by Empower™ 3 software.

### Amino acid analysis

The amino acid composition of soybean seeds was determined by an amino acid autoanalyzer (Model L-8900, Hitachi, Japan). In brief, 1.0 g samples were hydrolyzed with 10 ml of 6 M HCl for 24 h in Pyrex tubes for all amino acids, and then the hydrolysates were eluted through a concentration gradient starting at 0.2 M (pH 3.25) to 0.35 M (pH 5.25) at a rate of 0.4 ml/min. Amino acids were identified by an automatic amino acid analyzer equipped with an ion-exchange chromatography column. The total analysis time was 3.5 h per sample. The amino acid composition was reported in nmol/mg of protein powder on a dry weight basis. Each analysis was carried out for three replications.

### Preparation of 11S and 7S proteins

The 11S and 7S proteins were isolated according to the method of Yang et al. (2020). The 11S resultant solution was subsequently dialyzed against deionized (DI) water for 48 h in a dialysis tube (MW of 300,000 Da) and freeze dried to obtain the 11S fraction. Similarly, the 7S resultant solution was subsequently dialyzed against DI water for 48 h in a dialysis tube (MW 100,000 Da) and then freeze-dried to obtain the 7S proteins.

### SDS-polyacrylamide gel electrophoresis

SDS-polyacrylamide gel electrophoresis (SDS–PAGE) was performed according to the method of Thanh and Shibasaki (1976) with 4% stacking gels and 12% running gels. The protein samples were diluted to a final concentration of 0.1 mg/ml and solubilized in 0.125 M Tris–HCl buffer (pH 6.8) consisting of 1% (w/v) SDS, 2% (v/v) 2-mercaptoethanol (2-ME), 5% (v/v) glycerol and 0.025% (w/v) bromophenol blue and heated in a water bath (100°C) for 5 min before electrophoresis. For each sample, 10 μg was added to each lane in the stacking gel and electrophoresed. The gel was fixed in 50% trichloroacetic acid and then dyed with 0.25% Coomassie blue (R-250; Neuhoff et al., 1988), and the band intensities were analyzed with Image Lab software (Bio-Rad, Hercules, CA, United States).

### Fourier transform infrared spectroscopy

The 11S and 7S proteins were freeze-dried and characterized *via* a 100 FT-IR spectrometer (PerkinElmer, Akron, OH, United States). In brief, a mixture of 5 mg of sample and 500 mg of KBr powder was ground, pressed into a pellet and measured within the range of 400–4,000 cm^−1^ at a resolution of 4 cm^−1^ with 32 scans.

### Raman spectra

The Raman spectra of the 11S and 7S fractions were determined according to the modified method of Zhu et al. (2018). The 11S and 7S isolates were freeze-dried and spread on glass slides, followed by Raman experiments with an inVia Qontor Raman microscope (Renishaw, England). The samples were scanned in the measurement range of 400 cm^−1^ to 3,300 cm^−1^ under the following conditions: 3 scans, 3 s exposure time, 20× lens, and the use of a diaphragm with a 50 μm slit. The spectra were smoothed, and baselines were corrected and normalized against the phenylalanine band at 1003 cm^−1^.

### Circular dichroism analysis

Circular dichroism (CD) spectra of the 11S and 7S proteins were recorded using a circular dichroism analyzer (J-815, JASCO, Japan). The protein concentration was 0.2 mg/ml, and the path length was set to 0.1 mm. The spectra were measured in the far-UV region of 190–260 nm under the following conditions: a response time of 1 s and a bandwidth of 2 nm. The secondary structure contents of the 11S and 7S fractions were analyzed *via* the online Circular Dichroism Website.^1^

### Peak fitting and statistical analysis

The spectra were analyzed using OriginPro 2018 (OriginLab Corporation, Northampton, MA, USA). PeakFit v4.12 software was used to calculate the nonlinear fitting of the peaks in the spectral data. Baseline corrections were performed using a second derivative method for finding anchor points and detecting the baseline. Statistical analysis was performed by Student’s t tests at the level *p* = 0.01 and *p* = 0.05.

## Results

### Generation of transgenic soybean plants

**Figure 2:**
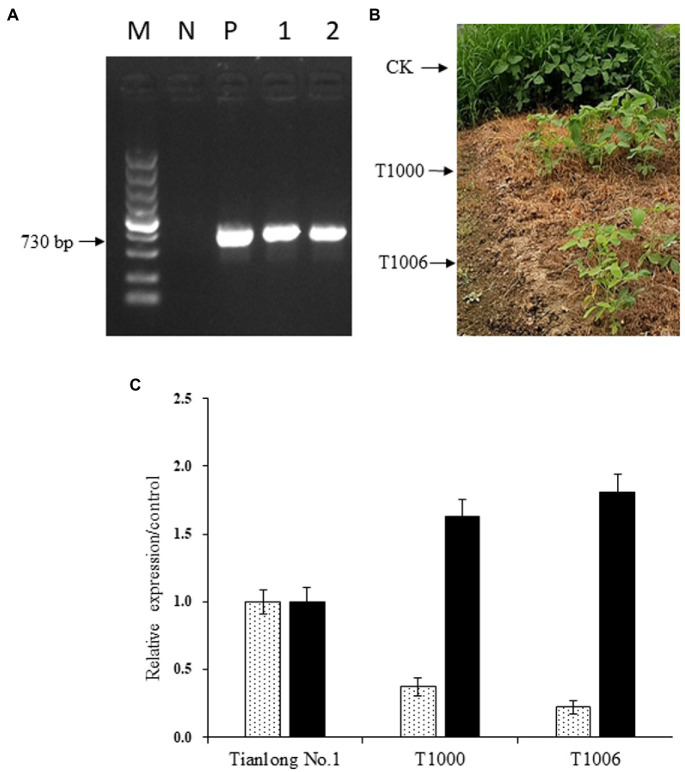
Expression of *OsEPSPS, CG-β-1* and *gy2* in transgenic soybean plants. **(A)** Detection of *OsEPSPS* transcripts by PCR amplification. M, DNA marker 5,000; N, the negative control ‘Tianlong No. 1’; P, the positive plasmid; Lane 1, T1000 transgenic lines; Lane 2, T1006 transgenic lines. **(B)** Glyphosate screening for transgenic lines. CK, ‘Tianlong No. 1’ without spraying glyphosate; T1000, T1000 transgenic lines sprayed with glyphosate (10 ml/l); T1006, T1006 transgenic lines sprayed with glyphosate (10 ml/l). **(C)** Detection of *CG-β-1* and *gy2 expression* using quantitative real-time PCR. CK, T1000, and T1006 represent ‘Tianlong No. 1’, T1000, and T1006 transgenic plants, respectively, and the levels of *CG-β-1* and *gy2* measured in ‘Tianlong No. 1’ were regarded as the control.

The cotyledonary node method was used to transform pCambia1300-OsEPSPS-RNAi-GmOle8pro-A2B1a constructs into soybean, and 102 T_0_ independent transgenic events were confirmed by both PCR analysis using *OsEPSPS* gene-specific primers and glyphosate (10 ml/l, v/v) screening. The next generation of transformants was validated *via* PCR and herbicide screening again and were grown in a greenhouse with 300 μmol m^−2^ s^−1^ illumination intensity and a 14/10 h photoperiod. Two independent transgenic events, T1000 and T1006, which showed high levels of *gy2* transcripts and a sharp reduction in *CG-β-1* expression according to PCR identification and displayed normal agronomic traits based on observations, were further advanced another four generations to obtain T_5_ plants (Figures 2A,B). Homozygous transgenic T_5_ plants were used to identify the expression levels of the *CG-β-1* and *gy2* genes by quantitative real-time PCR analysis with SYBR Green used as a reporter dye, and the actin gene served as an internal reference. The normalized expression levels of *CG-β-1* transcripts were downregulated sharply in the T1000 and T1006 independent transgenic events, with only 37 and 22% intensity in the R6 stage seeds compared with those of the nontransgenic control plants (‘Tianlong No. 1’), respectively. In contrast, the expression levels of *gy2* in the T1000 and T1006 lines harvested from R6 period seeds increased significantly (by 1.63- and 1.81-fold, respectively) compared with their original transcript levels (Figure 2C). Taken together, these results showed that the construct was integrated into the T1000 and T1006 genomes and displayed stable inheritance. Moreover, *CG-β-1* expression levels decreased dramatically, and the amount of *gy2* transcripts largely increased in both T1000 and T1006 plants.

### Accumulation of 7S globulins and 11S glycinin

**Figure 3:**
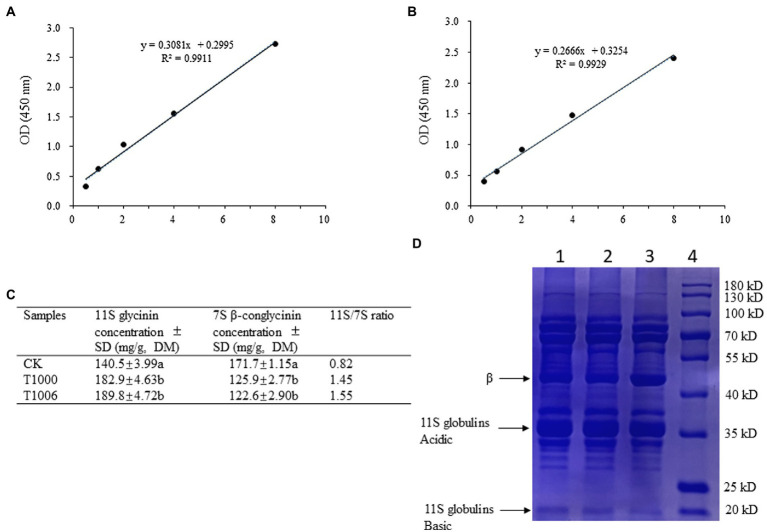
Analysis of the subunit composition of 7S β-conglycinin and 11S glycinin in soybean seeds. **(A,B)** are standard calibration curves of competitive ELISA for 7S and 11S globulins using polyclonal antibodies, respectively. **(C)** The content of 7S β-conglycinin and 11S glycinin in dry soybean seeds from different plants determined by ELISA results (3 replicates). The letters indicate the level of significance at the level *p* = 0.01. **(D)** Typical sodium dodecyl sulfate–polyacrylamide gel electrophoresis (SDS–PAGE, 12%) profiles of total proteins obtained from dry soybean seeds. The bands of the β subunit and the acidic and basic components of 11S globulins were noted. Lane 1, Lane 2, Lane 3, and Lane 4 represent T1000 and T1006 transgenic plants, ‘Tianlong No. 1’ and protein markers, respectively.

ELISAs were used to quantify the 7S and 11S fractions in the transgenic soybean seeds. The 11S protein contents of the T1000 and T1006 plants ranged from 182.9 to 189.8 mg/g dry seed flour, respectively, which were significantly higher than the values for the nontransformed controls (140.5 mg/g). The amount of the 7S globulins in the T1000 and T1006 lines was reduced substantially in transgenic seeds, with total seed weights ranging from 125.9 to 122.6 mg/g, respectively (Figures 3A–C). Further statistical analysis *via* t tests (*p* = 0.01) showed that there was a significant difference in the 7S and 11S globulin contents in seeds between the T1000 and T1006 lines and nontransgenic plants. The 11S/7S ratio increased significantly compared with its original value, from 0.82 to 1.45–1.55, and increased by 1.8 and 1.9 times in the T1000 and T1006 lines, respectively, compared to those of the transformation acceptor materials. We used Osborne fractions to extract the total protein (Osborne, 1924) and found that the 11S/7S ratio was also markedly improved in transgenic lines, although the value of 11S/7S was slightly different from the results obtained from Thanh and Shibasaki’s method (Thanh and Shibasaki, 1976; Supplementary Figures S1A–C). The contents of total seed protein harvested from continuous generations were also measured, and there was no significant difference in protein accumulation obtained from the seeds of T1000, T1006 and nontransgenic plants (Supplementary Table S2).

**Figure 4:**
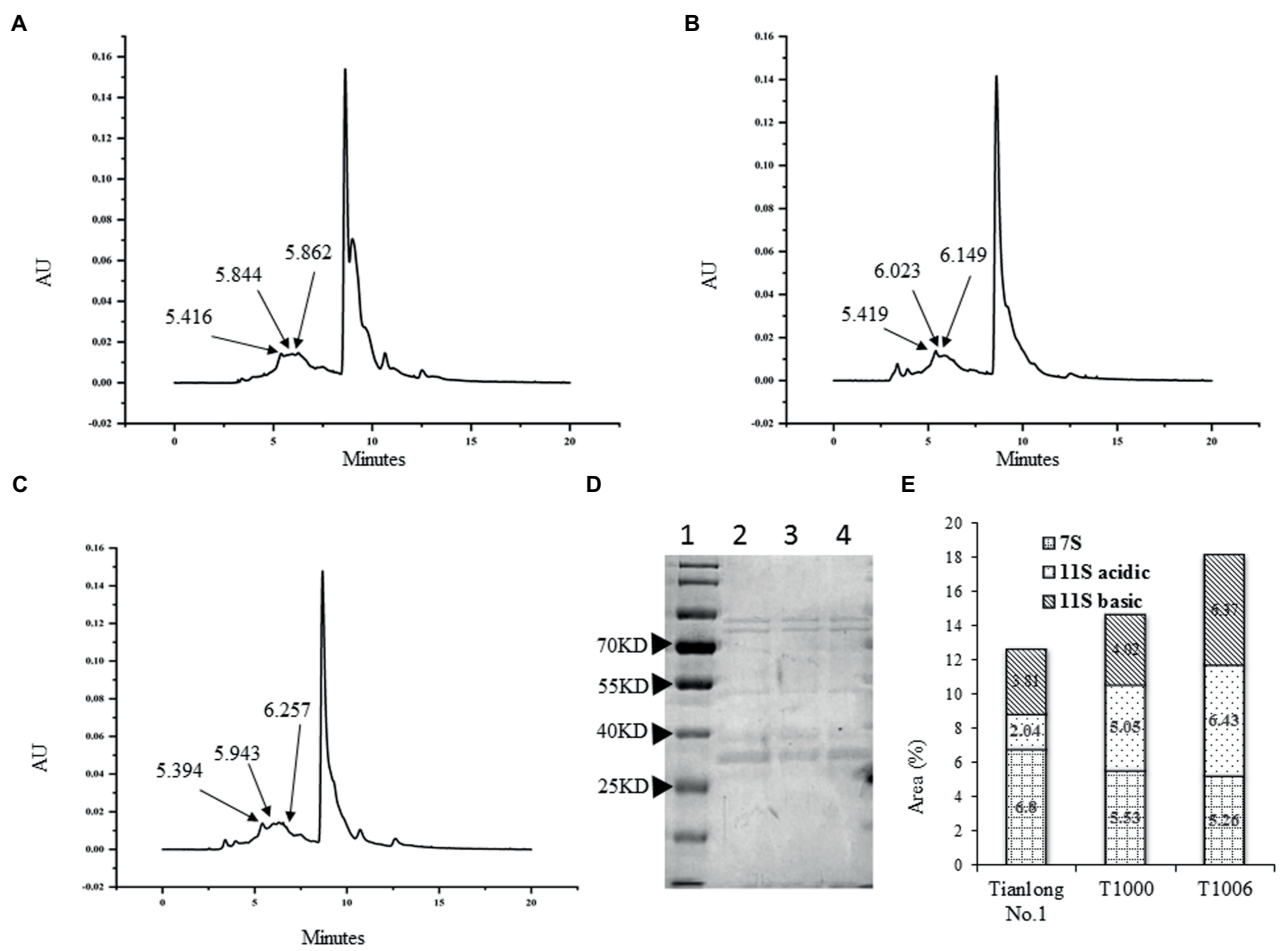
Gel permeation chromatography of SPI obtained from different plants. **(A–C)** are molecular weight (Mw) distribution profiles for the ‘Tianlong No. 1’, T1000, T1006 lines, respectively. **(D)** The proteins collected from elution times of 5.0–6.5 min were separated by SDS–PAGE. Lane 1, Lane 2, Lane 3, and Lane 4 represent the protein marker, T1000 lines, ‘Tianlong No. 1’, and T1006 transgenic plants, respectively. **(E)** The area distribution of 7S, 11S acidic, and 11S basic fractions from ‘Tianlong No. 1’, T1006 and T1000 plants, respectively.

SDS–PAGE showed that the electrophoretic band of the β subunit of 7S globulins obtained from the T1000 and T1006 lines was weaker than that of nontransgenic soybean seeds (Figure 3D). Under the same conditions, the A2B1a subunit in the protein profiles of 11S glycinin electrophoresis, which was separated into two fractions of approximately 38 and 19 kD, displayed stronger intensity in the transgenic T1000 and T1006 seeds than in the wild-type seeds (Figure 3D) and showed a similar electropherogram of the protein acquired from the Osborne fractions method (Supplementary Figure S1D). These results are consistent with the conclusions obtained from the ELISA data. Gel permeation chromatography (GPC) was performed to determine the protein size distribution and the 11S/7S ratio. As shown in Figures 4A–C, in the transgenic lines, the 11S soluble aggregates with slightly increased retention time had two peaks of 6.023 and 6.149 min for the T1000 lines and 5.943 and 6.257 min for the T1006 lines, respectively. Under the same elution conditions, there were 5.844 and 5.862 min peaks for 11S globulins in nontransgenic soybean plants. For 7S globulin, the profiles of soluble aggregates exhibited slight variability; there was a 5.419 min peak for the T1000 lines, the retention time was slightly longer than that of the transformation receptor (5.416 min), and the retention time (5.394 min) of the T1006 lines was slightly less than that of the control. The products collected from 5.0–6.5 elution time showed that the range of molecular weight contained α, α’, β and 11S acidic subunits resolved by SDS–PAGE (Figure 4D). The 11S/7S ratio was also calculated according to the area of the corresponding peak and was 2.43, 1.64, and 0.86 in T1006, T1000 and ‘Tianlong No. 1′ plants, respectively (Figure 4E). The 11S/7S ratio acquired from the peak area of the gel filtration chromatography was higher than the results from the ELISA analysis, which might be affected by other proteins with molecular weights similar to those of 7S and 11S globulins. However, the trend of the 11S/7S ratio in the T1006, T1000 and ‘Tianlong No. 1′ plants agreed with previous ELISA findings.

### The sulfur-amino acid content of transgenic soybean seeds significantly increases

The amino acid profile analysis of dry soybean seeds showed that there was a dramatic increase in cysteine and methionine content in T1000 and T1006 transgenic lines compared to wild-type plants, ‘Tianlong No. 1’ (Table 1). In T1000 and T1006 seeds, the cysteine content improved on average by 11.9 and 48.0%, respectively, while the methionine content was enhanced by 32.1 and 53.8%, respectively, when compared to nontransformed seeds. The total content of sulfur-containing amino acids was also markedly improved, amounting to 79.194 nmol/mg in dry soybean seeds from T1006 transgenic plants, 1.51 times higher compared to nontransformed controls (52.316 nmol/mg). Transgenic soybean by silencing *CG-β-1* expression *via* RNA interference and specifically enhancing the *gy2* transcripts in seeds displayed a significant accumulation of cysteine and methionine content, which was also consistent with the increase in the 11S/7S ratio in protein isolates obtained from T1000 and T1006 transgenic lines. In addition, the nonsulfur-containing amino acid composition of transgenic seed samples was only slightly different from that of nontransformed plants.

**Table 1:** The content of amino acids (nmol/mg) in dry soybean seeds from different lines.

Amino acid	Tianlong No. 1	T1000	T1006
Asp	453.691 ± 15.79a	432.28 ± 18.42a	475.247 ± 14.42a
Thr	169.241 ± 8.49a	172.302 ± 6.79a	179.234 ± 11.43a
Ser	249.984 ± 19.67a	234.098 ± 13.41a	263.007 ± 9.41a
Glu	657.252 ± 25.32a	588.476 ± 18.80b	673.612 ± 17.83a
Pro	227.855 ± 13.45a	211.335 ± 14.03a	239.334 ± 11.21a
Gly	277.724 ± 19.94a	275.355 ± 17.73a	287.952 ± 5.06a
Ala	236.177 ± 13.17a	230.895 ± 13.31a	245.875 ± 12.59a
**Cys**	**21.494 ± 0.27a**	**25.656 ± 0.54b**	**31.803 ± 0.34c**
Val	206.826 ± 7.08a	201.005 ± 16.61a	214.037 ± 1.58a
**Met**	**30.822 ± 0.42a**	**40.71 ± 0.36b**	**47.391 ± 0.27c**
Ile	182.711 ± 8.42a	167.186 ± 8.30a	186.300 ± 9.47a
Leu	298.154 ± 10.24a	276.46 ± 9.23a	304.812 ± 16.60a
Tyr	104.871 ± 3.98a	102.998 ± 5.39a	108.779 ± 4.93a
Phe	161.642 ± 5.95a	143.296 ± 6.29b	161.491 ± 4.02a
Lys	221.734 ± 6.94a	205.647 ± 7.52b	232.490 ± 4.69a
His	88.576 ± 6.21a	86.003 ± 8.29a	117.088 ± 7.99b
Arg	223.767 ± 13.98a	213.22 ± 14.11a	251.747 ± 10.01b

Data is expressed as the mean value with 3 replicates ± standard error; Values of the same line followed by the same letter are not significantly different at the level *p* = 0.05.Bold fonts indicate sulfur-containing amino acids.

### Secondary structures of the 11S and 7S isolates

**Figure 5:**
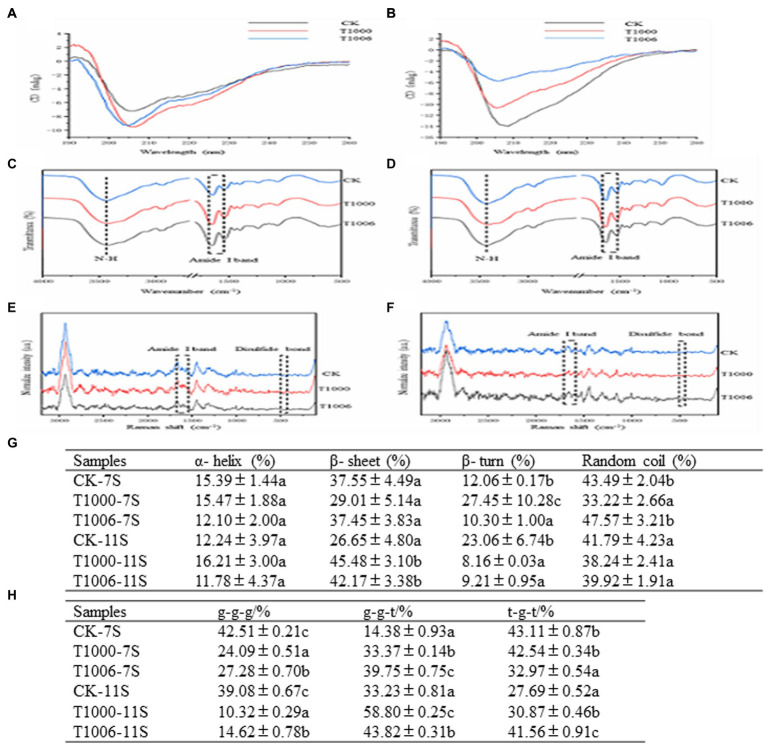
Circular dichroism, FTIR and Raman spectra of 7S and 11S isolates from different lines. **(A,B)** CD spectra; **(C,D)** FTIR spectra; **(E,F)** Raman spectra; CK, T1000, T1006 represent the ‘Tianlong No. 1’, T1000, T1006 lines, respectively. **(G)** Disulfide bond configuration of 7S and 11S fractions from different samples. Different letters indicate the level of significance at the level *p* = 0.05. **(H)** Secondary structure content of 7S and 11S fractions from different samples determined by CD, FTIR and Raman spectra. Different letters (a–c) indicate significant differences (*p* = 0.05).

Raman spectroscopy is a complement to infrared spectroscopy and is based on the inelastic scattering of light. The characteristic band of disulfide bonds is in the range of 500 to 550 cm^−1^. The Raman shifts of disulfide bonds are different in different vibration modes: 500–510 cm^−1^ is the gauche-gauche-gauche (g-g-g) mode; 525–535 cm^−1^ is the gauche-gauche-trans (g-g-t) mode; and 535–545 cm^−1^ is the trans-gauche-trans (t-g-t) mode. As shown in Figure 5G, the disulfide bonds in the 7S fractions from the nontransgenic lines are mainly associated with the two vibration modes of g-g-g and t-g-t, and their contents are 42.51 and 43.11%, respectively. In the 7S fractions obtained from the T1000 and T1006 lines, the modes of g-g-g are decreased and those of g-g-t are increased, whereas in the 11S isolates originating from T1000 and T1006 plants, the modes of g-g-g decreased sharply, and the modes of both g-g-t and t-g-t were obviously augmented.

The secondary structure was defined by far-ultraviolet circular dichroism, Raman spectroscopy and Fourier transform infrared spectroscopy (FTIR). The orientation and energy transition of peptide bonds in the secondary structure of proteins or peptides can be reflected by the CD spectrum in the far ultraviolet region. The characteristic CD peaks of the 7S and 11S isolates obtained from T1006, T1000 and ‘Tianlong No. 1’ are shown in Figures 5A,B. With respect to water-soluble proteins, the optically active substance exhibits anisotropy in light absorption in far-UV CD spectroscopy, and the secondary structure was identified based on the following: α-helices have absorption peaks at 208, 198 and 222 nm; β-sheets have absorption peaks at 195 and 216 nm; β-turns have absorption peaks at 205, 180–190 and 220–230 nm; and irregular curls have absorption peaks at 200 and 212 nm (Yang et al., 1986). There was a negative absorption peak at approximately 208 nm for the 7S isolates and a positive absorption peak for the 11S fractions.

The FTIR spectra of 11S and 7S in T1006, T1000 and ‘Tianlong No. 1’ plants are displayed in Figures 5C,D. The secondary structure of proteins can be quantitatively analyzed by means of the amide I band from Fourier transform infrared spectroscopy. According to the method of Qi et al. (2017), the corresponding secondary structures of proteins were calculated according to peak location attribution and peak area integral as follows: the α-helical structure ranged from 1,650 to 1,660 cm^−1^, and the parallel β-folded structures ranged from 1,618 cm^−1^ to 1,640 cm^−1^. The antiparallel β-folded structures ranged from 1,670 to 1,690 cm^−1^, the β-angle structure ranged from 1,660 cm^−1^ to 1,670 cm^−1^, and the random coiled structure ranged from 1,643 to 1,647 cm^−1^. The secondary structure of the proteins was also deduced from the amide I band of Raman spectroscopy. According to the relevant references of Raman spectroscopy (Yu et al., 2021), the characteristic vibration frequency of the α-helix structure in the protein amide I band ranges from 1,645–1,660 cm^−1^, the vibration frequency of the β-sheet ranges from 1,665–1,680 cm^− 1^, and the vibration frequency of the β-turn structure ranges from 1,680–1,690 cm^−1^ (Figures 5E,F). The secondary structure distribution of the 11S and 7S isolates in the T1006, T1000 and ‘Tianlong No. 1’ plants was determined according to the FTIR, Raman and CD spectroscopy results (Figure 5H). The contents of α-helices, β-sheets, β-turns and random coils isolated from ‘Tianlong No. 1’ materials were 15.39, 37.55, 12.06 and 43.49% for 7S isolates and 12.24, 26.65, 23.06, 41.79% for 11S isolates, respectively. For 7S isolates, the content of α-helices displayed slight variability in the T1000, T1006 and ‘Tianlong No. 1’ plants, ranging from 11.78–16.21%; however, the content of β-sheets in 11S isolates increased significantly, from 26.25 to 45.48%, and β-turns displayed the opposite trend, decreasing from 23.06 to 9.21%. The proportion of unordered coils ranged from 33.22 to 47.57%.

## Discussion

Efforts have been going to improve the levels of the essential sulfur-containing amino acids methionine and cysteine in soybean seeds, including traditional plant breeding, cultivation techniques, and transgenic engineering. Variations in the concentration of sulfur-containing amino acids in soybean protein can be influenced by the nitrogen source, the availability of reduced forms of sulfur, and environmental factors. A survey of commercial soybean varieties and resources in North America revealed unexpectedly high sulfur-containing amino acids and indicated that soybean amino acid composition can be affected by both geographic and environmental factors (Pfarr et al., 2018). To date, new cultivars with high SAA contents have not been reported, although several QTLs and candidate alleles associated with amino acid contents have been found (Panthee et al., 2006; Ramamurthy et al., 2014). S (MgSO_4_) application increased the proportion of Met + Cys in soybean seeds (Rushovich and WeilSulfur, 2021), and this required the correct application of S, which might be complex. Moreover, materials with high sulfur-containing amino acids have been influenced by environmental factors, nitrogen sources, and the availability of reduced forms of sulfur, which limits their use in field production. In this study, sulfur-containing amino acid content was significantly improved using *CG-β-1* RNA interference and the enhancement of A2B1a accumulation in seeds, and the level of Met + Cys maintained a stable ratio in various environmental factors.

Proteins rich in sulfur-containing amino acids, including synthetic methionine-rich proteins, MB-16, maize γ-zein proteins and delta-zein, have been successfully overexpressed in soybean to improve protein quality (Kim and Krishnan, 2004; Li et al., 2005; Zhang et al., 2014). However, the overall content of sulfur-containing amino acids in the transgenic soybean seeds increased only modestly, and it was not sufficient to meet the nutritional requirements of poultry or livestock. The insufficient accumulation of heterologous proteins in soybean typically accounts for approximately 1% of the total seed weight. In most cases, heterologous expression of sulfur-rich proteins in legumes is associated with a reduction in the accumulation of endogenous sulfur-rich seed proteins (Kim et al., 2014; Krishnan and Jez, 2018). Metabolic engineering of the sulfur assimilatory and methionine biosynthesis pathways has been applied to improve the cysteine and methionine contents of soybean. To date, cysteine biosynthesis OASSs, *Arabidopsis* cystathionine γ-synthase and ATP sulfurylase have been overexpressed in soybean (Song et al., 2013; Krishnan et al., 2018; Kim et al., 2020), and some encouraging results have been reported. Herein, metabolic engineering was adopted to improve the content of sulfur-containing amino acids, and no heterologous proteins were introduced into soybean by extremely increasing the 11S/7S ratio in seeds, which provides an attractive and precise engineering strategy to alter the functional and nutritional properties of soy protein.

Glycinin (11S) and β-conglycinin (7S) are the two main classes of multiple subunits of soybean seed storage proteins. Unfortunately, β-conglycinin is a major allergen for humans and is very deficient in sulfur-containing amino acids, especially the β subunit of 7S conglycinin (Herman, 2014). A2B1a subunit, which has the greatest abundance of sulfur-containing amino acids of all 11S glycinin fractions, cysteine and methionine account for 3 to 4.5% of the amino acid residues, similar to the levels of other high-quality dietary proteins (Paek et al., 1997). Hence, the glycinin fraction may be more desirable than β-conglycinin globulin in the production of foods with a well-balanced amino acid profile, and our study offered a very fine effective method to enhance the level of sulfur-containing amino acids in soybean seeds by raising the amount of 11S glycinin at the expense of reducing 7S globulin accumulation. There is a typical inverse relationship between 7S and 11S concentrations, and the glycinin content can be increased at the expense of β-conglycinin (Sui et al., 2021). Null mutations for each of the subunits of β-conglycinin were found to grow and reproduce normally, and the nitrogen content of seeds was similar to that of wild-type cultivars. Seeds of the induced mutant line appeared to compensate for the reduced nitrogen content by accumulating free amino acids (Takahashi et al., 2003) but had only a slight influence on the content of sulfur-containing amino acids. The total amino acid composition revealed a mild increase in transgenic soybean lines in which only β-conglycinin was knocked down (Kim et al., 2014), and in this study, the seed-specific promoter was introduced to enhance the accumulation of the A1B2a subunit in cooperation with reducing the total amount of β-subunits of 7S conglycinin, which increased the content of sulfur-rich amino acids to 79.194 nmol/mg and 51.38% higher than that of the wild-type control. This finding reflects a new promising approach to improve the level of sulfur-containing amino acids in dry soybean seeds. Moreover, the main agronomic traits, including the yield and the content of proteins, were not altered.

When the alpha helix content in protein molecules is low, the beta folding and random coil contents are relatively high, and the surface hydrophobicity increases (Chen et al., 2011). It was found that the alpha helix content in the secondary structure decreased and the beta folding and random coil content increased in 7S globulins from red bean (Tang and Sun, 2011). The content of sulfur-containing amino acids may reflect the ratio of 11S and 7S subunits (Zhang et al., 2018). Hydrophobic interactions, disulfide bonds and nondisulfide covalent bonds maintained the structure of the protein molecular network, and more sulfur-containing amino acids and β-sheets and increasing 11S/7S ratios were found to improve the textural qualities of Chiba tofu. Moreover, the correlation between the content of disulfide bonds and sulfur-containing amino acids (Met+Cys) was significantly positive (Zheng et al., 2021). The more β-sheets that are ordered, the denser the network structure of the protein gel and the greater the gel hardness (Zhao et al., 2008). In the present study, the amount of the A1B2a subunit was sharply improved compared with its normal value in common 11S isolates, which was a new formula for 11S glycinin. Similarly, reducing the ratio of the β-subunit of 7S conglycinin would generate other kinds of 7S globulins, which is different from ordinary 7S isolates. Our results showed that the content of sulfur-containing amino acids in the T1000 and T1006 lines was markedly improved, and the ratio of 11S/7S and β-sheets was also increased. As such, we provided a new soybean material for use in the production of foods with a well-balanced amino acid profile.

In summary, the present study demonstrated that sulfur-containing amino acid content was significantly improved using *CG-β-1* RNA interference and the enhancement of A2B1a accumulation in soybean seeds. The new formulae of the 7S and 11S fractions underwent conformational transitions accompanied by a decrease in the α-helix content and a corresponding increase in the β-sheet content.

## Data availability statement

The datasets presented in this study can be found in online repositories. The names of the repository/repositories and accession number(s) can be found in the article/Supplementary material.

## Author contributions

BW performed spectra and data analysis. DT conducted experiments of PCR identification and FTIR. CY performed amino acid analysis. LY conducted SDS-PAGE and ELISA experiments. XM constructed the vector. TW carried out soybean transformation and screening in the field. All authors contributed to the article and approved the submitted version.

## Funding

This work was supported by grants from National Science and Technology Major Project (2016ZX08004-003), National Natural Science Foundation of China (31871645), and Shanghai Collaborative Innovation Center of Agri-seeds (ZXWH2150201/008).

## Conflict of interest

The authors declare that the research was conducted in the absence of any commercial or financial relationships that could be construed as a potential conflict of interest.

## Publisher’s note

All claims expressed in this article are solely those of the authors and do not necessarily represent those of their affiliated organizations, or those of the publisher, the editors and the reviewers. Any product that may be evaluated in this article, or claim that may be made by its manufacturer, is not guaranteed or endorsed by the publisher.

## Footnotes

^1^ http://dichroweb.cryst.bbk.ac.uk

## Supplementary material

Supplementary Figure S1 Analysis of subunit composition of 7S β-conglycinin and 11S glycinin in soybean seeds determined by Osborne fractions method. **(A,B)** are standard calibration curves of competitive ELISA for 7S and 11S globulins using polyclonal antibody respectively. **(C)** The content of 7S β-conglycinin and 11S glycinin in dry soybean seeds from different plants determined by ELISA results (3 replicates); The letters indicate the level of significance at the level *P* = 0.01. **(D)** Typical sodium dodecyl sulphate-polyacrylamide gel electrophoresis (SDS-PAGE, 12%) profiles of total proteins obtained from dry soybean seeds. The band of β subunit, the acidic and basic components of 11S globulins were noted respectively; Lane 1, lane 2, lane 3, lane 4 represented ‘Tianlong No. 1’, T1000, T1006 transgenic plants and protein marker respectively.
