# Supplementary material for: Increased sulfur-containing amino acid content and altered conformational characteristics of soybean proteins by rebalancing 11S and 7S compositions
Source: Front Plant Sci. 2022 Sep 2;13:828153. doi: 10.3389/fpls.2022.828153 (PMC9478179; doi:10.3389/fpls.2022.828153)
Supplement: Supplementary file 1 [file Table_1.DOCX]

Table S1

The sequences of primers used in the experiment

| Items | Target genes | Sequences (5’-3’) |
| --- | --- | --- |
| q7S-F | *CG-β-1* | GCGGTTTCCTTTGTTGGTGTTGCTG |
| q7S-R |  | CGAATGCGACCGTTTTGGTTCTCAA |
| q11S-F | *gy2* | CATGGTGGATGTACAACAATGAAGA |
| q11S-R |  | GCTGCTGCTGATATTTTAGAAACTC |
| epsps-F | *OsEPSPS* | ATGCAACTTATGTGCTTGATGGAG |
| epsps-R |  | GTTTCCTTT ACTCTCCAGG AAGCC |
| actin-F | actin | AGATAGGGAAATTGTGCAGG |
| actin-R |  | CTAATGGCAATTGCAGCTCTC |
