# Supplementary material for: Increased sulfur-containing amino acid content and altered conformational characteristics of soybean proteins by rebalancing 11S and 7S compositions
Source: Front Plant Sci. 2022 Sep 2;13:828153. doi: 10.3389/fpls.2022.828153 (PMC9478179; doi:10.3389/fpls.2022.828153)
Supplement: Supplementary file 2 [file Table_2.DOCX]

Table S2

Investigation for Agronomic traits of Tianlong No. 1, T1000 and T1006 materials in 2019-2020

| Year | Name | Growth period （day） | Leaf length (mm) | Leaf width (mm) | Flower color | Pubescence color | Podding habit | Canopy  character | Pod height (cm) | plant height (cm) | **nod number in main stem ，** | Branch number | Pod shattering (%) | Pods per plant | Seed weight per plant (g) | 100-seed weight (g) | Yield (g/6.7m^3^) | protein content (%) | oil content (%) |
| --- | --- | --- | --- | --- | --- | --- | --- | --- | --- | --- | --- | --- | --- | --- | --- | --- | --- | --- | --- |
| 2019 | Tianlong No. 1 | 98 | 157.2±7.3a | 82.6±4.5a | white | white | determinate | compact | 9.87±1.5a | 57.3±6.9a | 13.7±2.1a | 2.3±0.5a | 0.02 | 28.8±3.3a | 14.1±2.1a | 24.2±1.9a | 2115.5±78 | 41.64 | 20.11 |
|  | T1000 | 98 | 156.9±8.2a | 82.9±4.9a | white | white | determinate | compact | 10.01±1.1a | 57.2±7.3a | 13.7±2.3a | 2.4±0.4a | 0.01 | 28.9±3.1a | 14.2±2.5a | 24.2±2.3a | 2119.1±89 | 41.89 | 20.09 |
|  | T1006 | 98 | 157.4±7.9a | 82.8±4.1a | white | white | determinate | compact | 10.13±1.3a | 57.3±5.4a | 13.8±3.1a | 2.2±0.4a | 0.01 | 28.7±3.5a | 14.4±2.3a | 24.3±2.7a | 2120.7±99 | 41.93 | 20.08 |
| 2020 | Tianlong No. 1 | 99 | 160.7±8.7a | 83.2±3.9a | white | white | determinate | compact | 10.11±1,6a | 60.5±6.7a | 14.1±2.7a | 2.3±0.3a | 0.01 | 29.2±4.0a | 14.9±3.2a | 25.8±2,.5a | 2243.8±90 | 42.34 | 19.93 |
|  | T1000 | 99 | 161.4±8.3a | 82.8±4.6a | white | white | determinate | compact | 10.20±1.6a | 60.7±6.9a | 13.9±3.7a | 2.3±0.4a | 0.01 | 29.4±3.9a | 15.1±3.3a | 26.1±2.8a | 2251.0±79 | 42.55 | 19.88 |
|  | T1006 | 99 | 161.1±9.1a | 83.5±5.2a | white | white | determinate | compact | 10.17±1.9a | 60.3±2.5a | 14.3±3.4a | 2.4±0.5a | 0.01 | 29.3±3.7a | 15.2±3.8a | 26.2±2.7a | 2249.9±86 | 42.60 | 19.87 |

Note: Data represented the mean value of 10 samples with 3 replicates; Values followed by the same letter are not significantly different at the level P=0.05.
