# Supplementary figures and images for: Increased sulfur-containing amino acid content and altered conformational characteristics of soybean proteins by rebalancing 11S and 7S compositions
Source: Front Plant Sci. 2022 Sep 2;13:828153. doi: 10.3389/fpls.2022.828153 (PMC9478179; doi:10.3389/fpls.2022.828153)

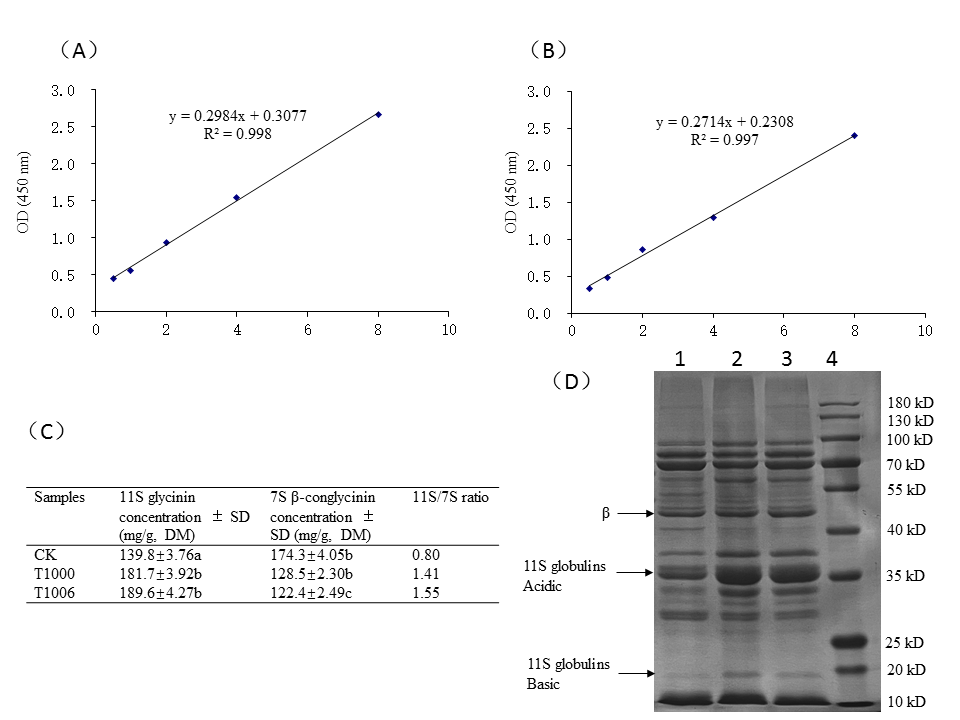

Supplement: Supplementary Figure S1 — Analysis of subunit composition of 7S β-conglycinin and 11S glycinin in soybean seeds determined by Osborne fractions method. (A,B) are standard calibration curves of competitive ELISA for 7S and 11S globulins using polyclonal antibody respectively. (C) The content of 7S β-conglycinin and 11S glycinin in dry soybean seeds from different plants determined by ELISA results (3 replicates); The letters indicate the level of significance at the level P = 0.01. (D) Typical sodium dodecyl sulphate-polyacrylamide gel electrophoresis (SDS-PAGE, 12%) profiles of total proteins obtained from dry soybean seeds. The band of β subunit, the acidic and basic components of 11S globulins were noted respectively; Lane 1, lane 2, lane 3, lane 4 represented ‘Tianlong No. 1’, T1000, T1006 transgenic plants and protein marker respectively. [file Image_1.TIF]
